# LncRNA-MIAT activates hepatic stellate cells via regulating Hippo pathway and epithelial-to-mesenchymal transition

**DOI:** 10.1038/s42003-023-04670-z

**Published:** 2023-03-18

**Authors:** Yating Zhan, Qiqi Tao, Qishan Meng, Rongrong Zhang, Lifan Lin, Xinmiao Li, Lei Zheng, Jianjian Zheng

**Affiliations:** 1 Key Laboratory of Diagnosis and Treatment of Severe Hepato-Pancreatic Diseases of Zhejiang Province, The First Affiliated Hospital of Wenzhou Medical University, Wenzhou, 325000 China; 2 Key Laboratory of Clinical Laboratory Diagnosis and Translational Research of Zhejiang Province, The First Affiliated Hospital of Wenzhou Medical University, Wenzhou, 325000 China; 3 Laboratory Medicine Center, Nanfang Hospital, Southern Medical University, Guangzhou, 510515 China

**Keywords:** Mechanisms of disease, Cell death

## Abstract

Long non-coding RNA-myocardial infarction-associated transcript (lncRNA-MIAT) has been reported to play an important role in the development of multiple cancers. However, the biological roles of MIAT in liver fibrosis are still unknown. In this study, the expression of MIAT is up-regulated during liver fibrosis. Silencing MIAT leads to the suppression of hepatic stellate cell (HSC) proliferation and collagen expression. Double immunofluorescence analysis additionally demonstrates that MIAT inhibition leads to the suppression of type I collagen and α-SMA in vitro. In vivo, MIAT knockdown contributes to the inhibition of fibrosis progression and collagen accumulation. MIAT is confirmed as a target of miR-3085-5p, and the co-location of MIAT and miR-3085-5p is found in HSC cytoplasm. Interestingly, there is a negative correlation between MIAT expression and miR-3085-5p level in cirrhotic patients as well as activated HSCs. In addition, the effects of MIAT inhibition on HSC inactivation are blocked down by miR-3085-5p inhibitor. YAP is a target of miR-3085-5p. Reduced YAP caused by loss of MIAT is reversed by miR-3085-5p inhibitor. Notably, YAP knockdown results in the suppression of MIAT-mediated epithelial-to-mesenchymal transition (EMT) process. In conclusion, we demonstrate that MIAT enhances the activation of HSCs, at least in part, via miR-3085-5p/YAP/EMT signaling pathway.

## Abstract (web summary)

LncRNA MIAT is shown to be a potential clinical biomarker for liver fibrosis and enhances hepatic stellate cell activation via miR-30855p/YAP/EMT signalling.

## Introduction

Liver fibrosis is a wound-healing response to chronic liver injury caused by multiple factors such as hepatophilic virus infection, excessive alcohol consumption, and non-alcoholic fatty liver disease^1,2^. Without effective therapy, liver fibrosis will ultimately evolve to cirrhosis and even hepatocellular carcinoma (HCC). Liver fibrosis is characterized by excessive deposition of extracellular matrix (ECM) derived from activated hepatic stellate cells (HSCs)^3^. Besides, myofibroblasts transdifferentiated by HSCs contribute to the production of a series of profibrogenic factors such as transforming growth factor-β, connective tissue growth factor, leading to the accumulation of ECM^4^. Therefore, activation of HSCs is crucial for the progression of liver fibrosis and targeting HSC activation treatment may be a promising therapeutic strategy for liver fibrosis.

Long non-coding RNAs (lncRNAs) are longer than 200 nt RNA transcripts with no protein-encoding functions^5^. It has been reported that lncRNAs can regulate transcription and/or translation through directly combining with DNA, RNA or proteins^6^. Besides, a growing body of evidence has demonstrated that lncRNAs participate in the regulation of diverse cellular processes, including cell cycle, differentiation and metabolism^7^. For instance, myocardial infarction-associated transcript (MIAT) promotes proliferation, migration and invasion in papillary thyroid carcinoma cells^8^. Increasing evidence has also shown the importance of MIAT in organ fibrosis such as atrial fibrosis and renal interstitial fibrosis^9,10^. However, the biological functions of MIAT in liver fibrosis are still unclear.

## Results

### Increased expression of MIAT in the livers from patients with cirrhosis

**Fig. 1:**
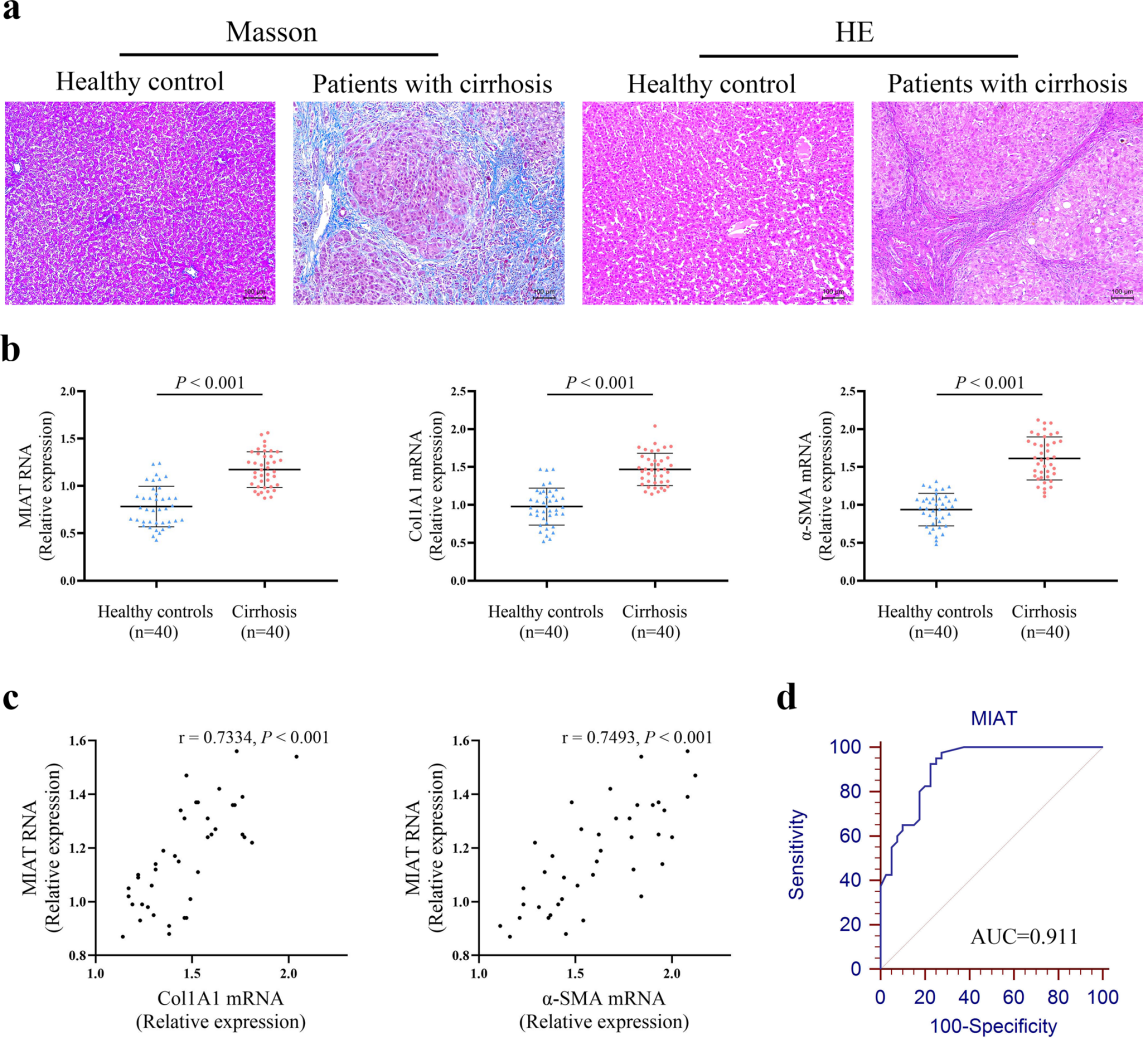
Expression of MIAT in patients with cirrhosis. **a** Masson and HE staining. Scale bar, 100 μm. **b** Expressions of MIAT, Col1A1 and α-SMA in the livers from healthy control (*n* = 40) and cirrhotic patients (*n* = 40). **c** Correlation between MIAT expression and Col1A1 as well as α-SMA (*n* = 40). **d** ROC curve of MIAT for diagnosing liver fibrosis. Each value is the mean ± SD of three independent experiments.

To determine whether MIAT is dysregulated in liver fibrosis progression, the expression of MIAT was examined in the liver tissues of patients with cirrhosis. Results of HE showed that the normal liver tissues were destroyed and replaced by new nodules (Fig. 1a). Masson staining revealed collagen accumulation in cirrhotic tissues (Fig. 1a). As shown in Fig. 1b, the mRNA expressions of alpha-1(I) collagen (Col1A1) and α-smooth muscle actin (α-SMA) were up-regulated in patients with cirrhosis. In addition, there was an increase in MIAT level in human cirrhotic tissues compared with the control (Fig. 1b). Interestingly, MIAT expression in patients with cirrhosis was positively correlated with Col1A1 mRNA expression (*r* = 0.7334, *p* < 0.001, Fig. 1c). Similarly, MIAT expression was positively correlated with α-SMA mRNA expression (*r* = 0.7493, *p* < 0.001, Fig. 1c). We also investigated whether liver MIAT could be a potential biomarker for diagnosing liver fibrosis. Results of receiver operating characteristic (ROC) curve indicated that MIAT had a good performance in the diagnosis of liver fibrosis, with an area under curve (AUC) value of 0.911 [95% confidence interval (CI), 0.826–0.963] (Fig. 1d). Sensitivity was 92.5% and specificity was 77.5% at the cutoff value of 0.92. These data indicate that MIAT may play a crucial role in the development of liver fibrosis.

### Loss of MIAT suppresses HSC proliferation and activation

**Fig. 2:**
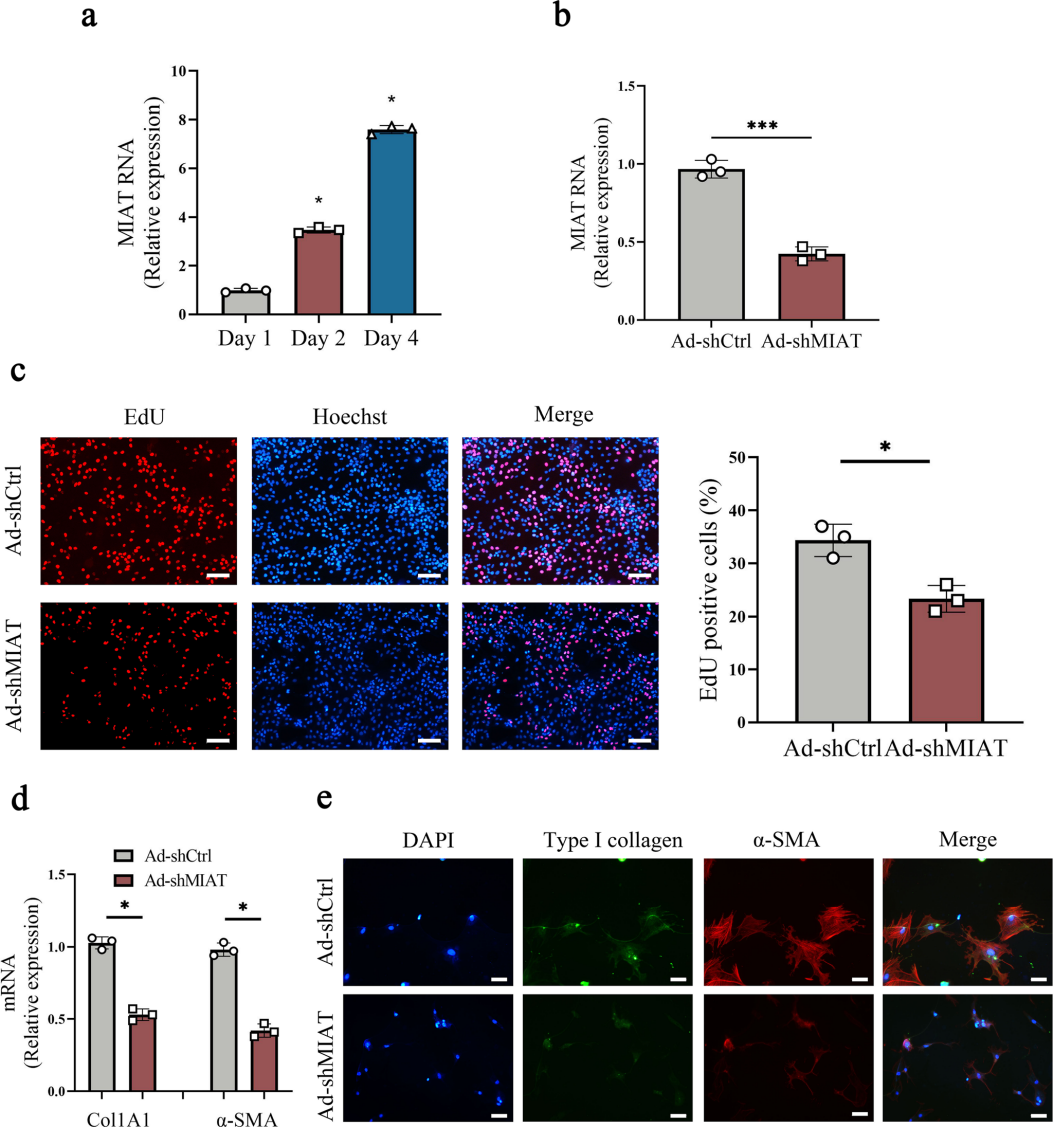
Loss of MIAT promotes the suppression of HSC proliferation and activation. **a** MIAT expression in primary HSCs at day 1, 2 and 4 (*n* = 3 per group). **b** MIAT expression in primary HSCs transduced with Ad-shMIAT (*n* = 3 per group). **c** EdU assay in HSCs with Ad-shMIAT treatment (*n* = 3 per group). Scale bar, 50 μm. **d** Col1A1 and α-SMA expressions (*n* = 3 per group). **e** Immunofluorescence staining for Type I collagen (green) and α-SMA (red) in HSCs with Ad-shMIAT treatment. Scale bar, 20 μm. Each value is the mean ± SD of three independent experiments. *p < 0.05, ***p < 0.001 compared with the control.

Expression of MIAT in primary HSCs isolated from healthy mice was additionally examined. It was found that MIAT was gradually up-regulated in primary HSCs during cell culture (Fig. 2a). To test the hypothesis that MIAT participates in liver fibrosis progression, HSCs were treated with adenoviral vectors expressing short hairpin RNA against MIAT (Ad-shMIAT) to silence MIAT level. Clearly, MIAT was significantly reduced by Ad-shMIAT in cells (Fig. 2b). During the period of HSC activation, transdifferentiation of HSCs is generally with many characteristics such as increased proliferation, over-expressed α-SMA and enhanced collagen. Results of Ethynyl deoxyuridine (EdU) assays indicated that loss of MIAT reduced HSC proliferation (Fig. 2c). In addition, results of wound healing showed that HSC migration was inhibited by MIAT knockdown (Fig. S1). MIAT inhibition also attenuated HSC collagen accumulation. Reduced level of Col1A1 mRNA was found in Ad-shMIAT group (Fig. 2d). Similarly, immunofluorescence results of type I collagen showed a reduction in the level of green fluorescence (Fig. 2e). In line with it, Ad-shMIAT led to the inhibition of α-SMA mRNA level (Fig. 2d). Immunofluorescence analysis further confirmed the reduction in α-SMA protein actin fibers (Fig. 2e). As shown by double immunofluorescence analysis, enhanced type I collagen and α-SMA in activated HSCs were suppressed by MIAT knockdown. Therefore, MIAT may act as a pro-fibrotic factor in HSC activation.

### MIAT knockdown ameliorates the severity of liver fibrosis caused by carbon tetrachloride (CCl_4_)

**Fig. 3:**
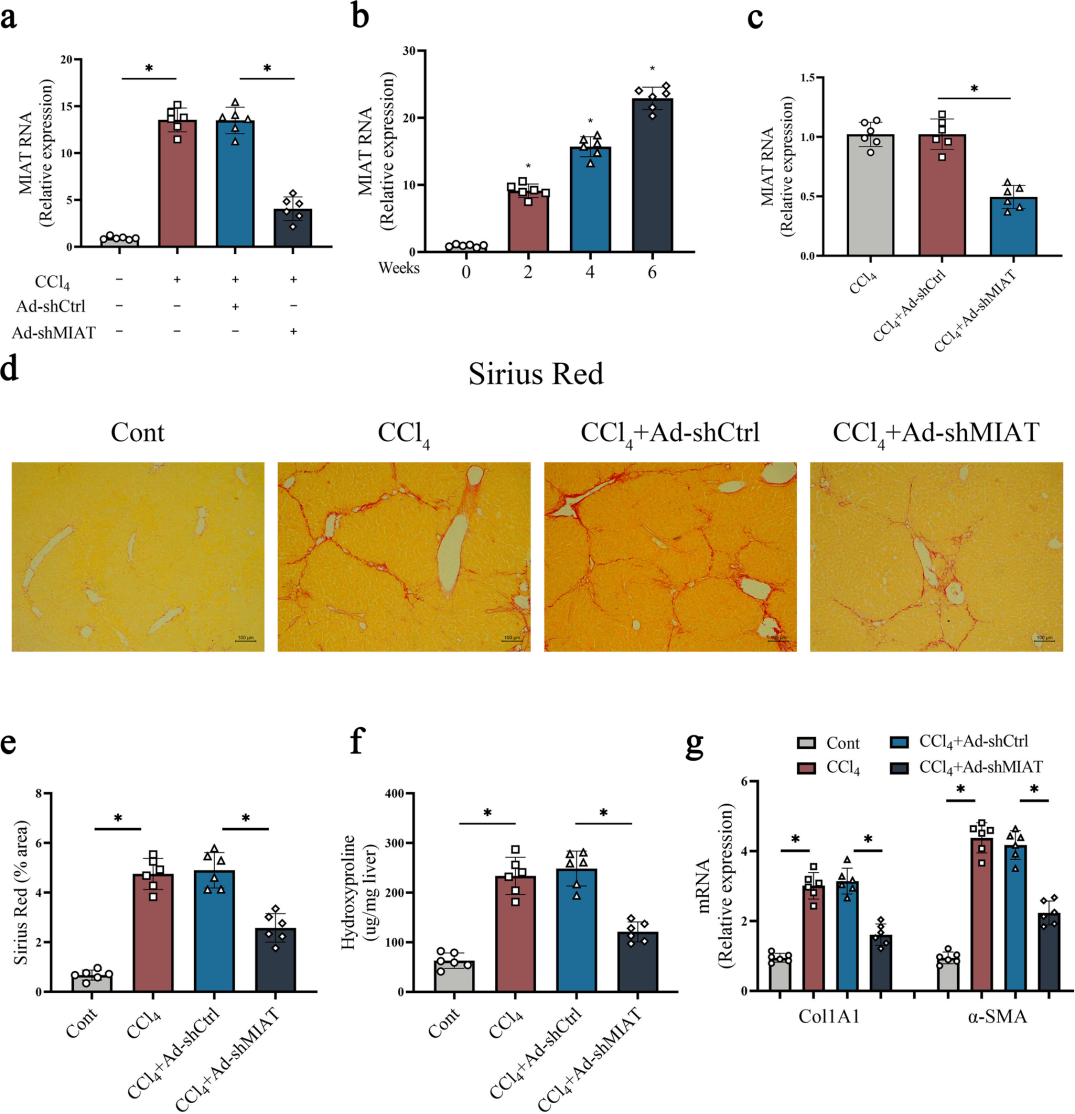
MIAT knockdown ameliorates the severity of liver fibrosis caused by CCl_4_. **a** MIAT expression in the livers of CCl_4_ mice treated with Ad-shMIAT (*n* = 6 per group). **b** MIAT expression in isolated HSCs from CCl_4_ mice (*n* = 6 per group). **c** MIAT expression in isolated HSCs from CCl_4_ mice with Ad-shMIAT treatment (*n* = 6 per group). **d**, **e** Sirius red staining and statics analysis (*n* = 6 per group). Scale bar, 100 μm. **f** Hydroxyproline level (*n* = 6 per group). **g** Col1A1 and α-SMA expressions (*n* = 6 per group). Each value is the mean ± SD of six independent experiments. *p < 0.05.

CCl_4_ has been reported to be a classic liver injury inducer^11^. In this study, CCl_4_ was used for the construction of liver fibrosis mice model. Consistent with the detection in vitro, MIAT was also up-regulated in the livers from CCl_4_ mice (Fig. 3a). Then, primary HSCs were isolated from mice treated with CCl_4_. It was found that MIAT was gradually enhanced at a time-dependent manner during CCl_4_ treatment (Fig. 3b). Clearly, reduced MIAT could be found in Ad-shMIAT-treated CCl_4_ mice as well as their isolated primary HSCs (Fig. 3a, c). Whether MIAT knockdown has an inhibitory effect on liver fibrosis in vivo was subsequently explored. Analysis of Sirius Red staining demonstrated that MIAT knockdown led to the suppression of collagen deposits induced by CCl_4_ (Fig. 3d, e). Likewise, hydroxyproline caused by CCl_4_ was inhibited by MIAT knockdown (Fig. 3f). Additionally, there was a reduction in collagen expression as well as α-SMA level in CCl_4_ mice treated with Ad-shMIAT (Fig. 3g). Our data suggest that knockdown of MIAT inhibits liver fibrosis progression in vivo.

### microRNA-3085-5p (miR-3085-5p) is a target of MIAT

**Fig. 4:**
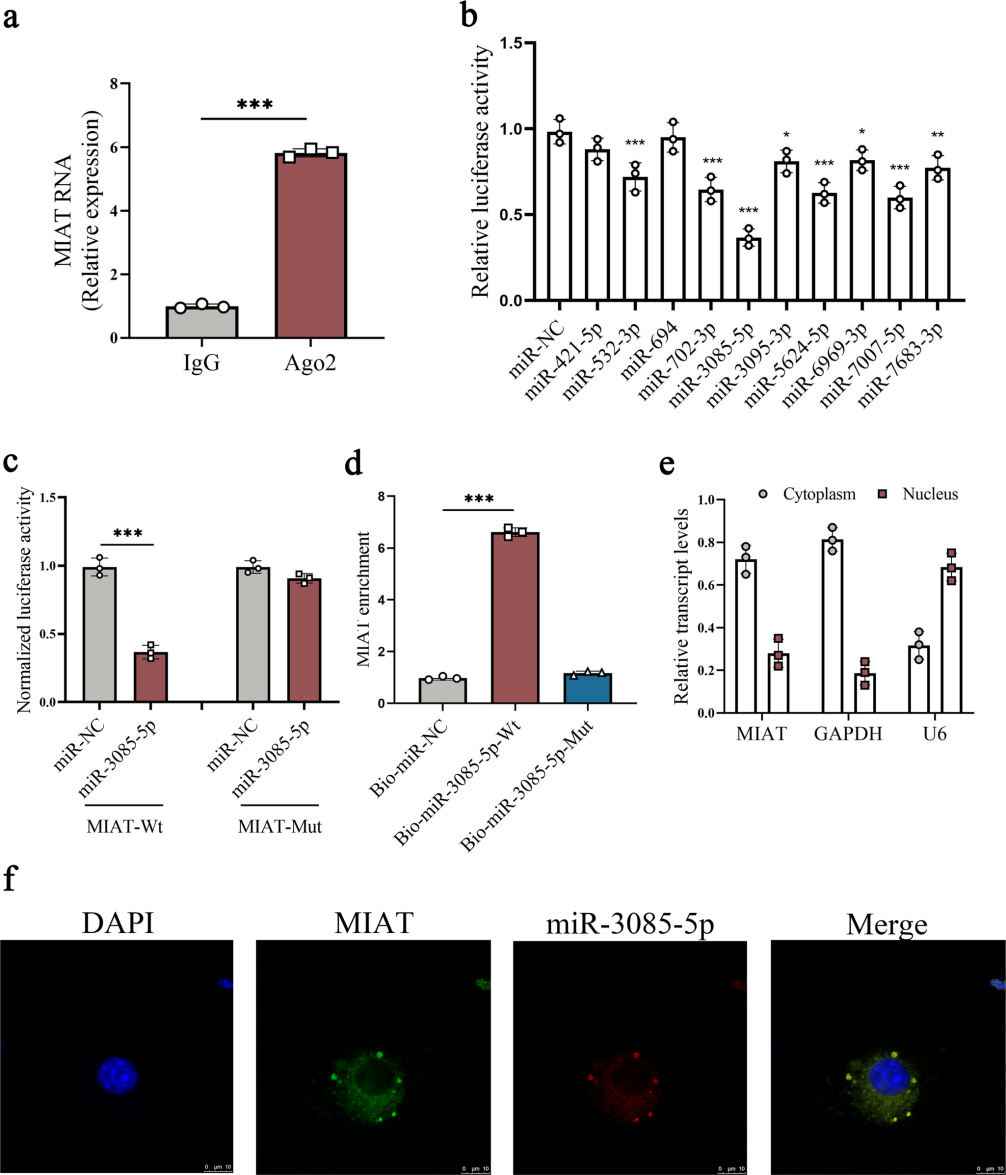
miR-3085-5p is a target of MIAT. **a** RIP experiments using Ago2 antibody (*n* = 3 per group). **b** Luciferase assays of pmirGLO-MIAT-Wt in HEK-293T cells with miR-NC or 10 miRNAs (*n* = 3 per group). **c** Luciferase assays of pmirGLO-MIAT-Wt or pmirGLO-MIAT-Mut in HEK-293T cells with miR-NC or miR-3085-5p (*n* = 3 per group). **d** Pull-down assay for the interaction between MIAT and miR-3085-5p (*n* = 3 per group). **e** Distribution of MIAT expression in the cytoplasm and nucleus in HSCs (*n* = 3 per group). **f** FISH analysis for co-localization between miR-3085-5p and MIAT. Scale bar, 10 μm. Each value is the mean ± SD of three independent experiments. *p < 0.05; **p < 0.01; ***p < 0.001 compared with the control.

A recent study demonstrated that lncRNAs act as miRNA sponges, resulting in the restoration of depressed miRNA targets^12^. Next, we analyzed whether MIAT could act as a miRNA sponge. As confirmed by results of RNA immunoprecipitation (RIP), MIAT was obviously up-regulated in the Ago2 group in comparison with the IgG group (Fig. 4a), suggesting the potential ability of MIAT in binding Ago2 and miRNAs. Bioinformatic analysis was performed to predict potential miRNAs that may bind to MIAT via miRanda and RNAhybrid. Finally, miRNAs including miR-421-5p, miR-532-3p, miR-694, miR-702-3p, miR-3085-5p, miR-3095-3p, miR-5624-5p, miR-6969-3p, miR-7007-5p, and miR-7683-3p were predicted to bind MIAT. Then, whether MIAT could effectively bind the above miRNAs was examined via luciferase activity assays. Results of luciferase activity assays indicated that the activities of the luciferase reporter were inhibited by most miRNAs, with the lowest in miR-3085-5p (Fig. 4b). Thus, miR-3085-5p was selected for the subsequent studies. We mutated the miR-3085-5p recognition sequences on MIAT (Fig. 4c). It was confirmed that miR-3085-5p had an effect on luciferase activity of MIAT-Wild Type (MIAT-Wt), whereas MIAT-Mutant Type (MIAT-Mut) not (Fig. 4c). Furthermore, miR-3085-5p interaction with MIAT was confirmed by pull-down assays. Pull-down results showed that MIAT enrichment was significantly higher in biotinylated miR-3085-5p-Wild Type (bio-miR-3085-5p-Wt) group (Fig. 4d). By contrast, biotinylated miR-3085-5p-Mutant Type (bio-miR-3085-5p-Mut) group had no effect on MIAT enrichment. Further studies were performed to determine the distribution of MIAT in HSCs. As indicated by Fig. 4e, MIAT was mainly expressed in the cytoplasm in primary HSCs. In addition, miR-3085-5p and MIAT were found to be co-located in the cytoplasm by fluorescence in situ hybridization (FISH) analysis (Fig. 4f). Taken together, miR-3085-5p is a target of MIAT.

### Effect of MIAT inhibition on HSC inactivation is associated with miR-3085-5p

**Fig. 5:**
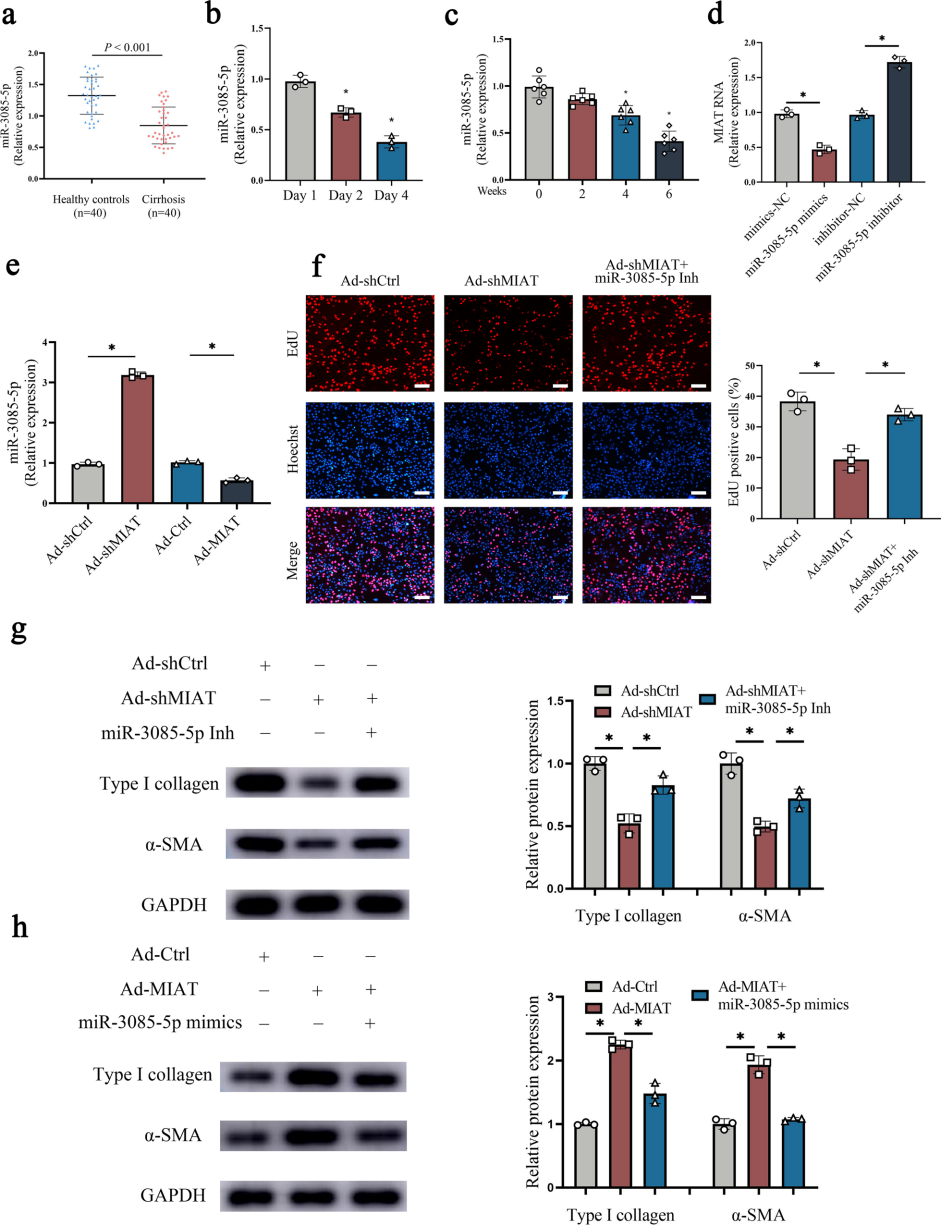
miR-3085-5p is involved in the effects of MIAT in HSC activation. **a** Expression of miR-3085-5p in healthy control (*n* = 40) and cirrhotic patients (*n* = 40). **b** miR-3085-5p expression in primary HSCs at day 1, 2 and 4 (*n* = 3 per group). **c** miR-3085-5p expression in isolated HSCs from CCl_4_ mice (*n* = 6 per group). **d** MIAT expression in primary HSCs with miR-3085-5p mimics or inhibitor treatment (*n* = 3 per group). **e** miR-3085-5p expression in primary HSCs transfected with Ad-shMIAT or Ad-MIAT (*n* = 3 per group). **f** Cell proliferation in HSCs transfected with Ad-shCtrl, Ad-shMIAT or Ad-shMIAT plus miR-3085-5p inhibitor (*n* = 3 per group). Scale bar, 50 μm. **g** Protein expressions of Type I collagen and α-SMA in HSCs transfected with Ad-shCtrl, Ad-shMIAT or Ad-shMIAT plus miR-3085-5p inhibitor (*n* = 3 per group). **h** Protein expressions of Type I collagen and α-SMA in HSCs transfected with Ad-Ctrl, Ad-MIAT or Ad-MIAT plus miR-3085-5p mimics (*n* = 3 per group). Each value is the mean ± SD of three independent experiments. *p < 0.05.

To determine whether the effect of MIAT inhibition on HSC inactivation is associated with miR-3085-5p, the expression of miR-3085-5p was examined during liver fibrosis in vivo and in vitro. MiR-3085-5p down-regulation was shown in the fibrotic tissues from patients with cirrhosis (Fig. 5a). MiR-3085-5p level was also reduced in activated HSCs (Fig. 5b). In line with these, miR-3085-5p was additionally down-regulated in HSCs isolated from CCl_4_ mice at different weeks (Fig. 5c). All these data suggest that MIAT was negatively correlated with miR-3085-5p. In addition, over-expression of miR-3085-5p down-regulated MIAT while loss of miR-3085-5p up-regulated MIAT (Fig. 5d). Likewise, MIAT upregulation resulted in the reduction in miR-3085-5p, whereas MIAT knockdown led to the increase in miR-3085-5p (Fig. 5e). It is still unknown whether miR-3085-5p has an impact on the activation of HSCs. As shown in Fig. S2a, the treatment of miR-3085-5p overexpression suppressed HSC proliferation, while the treatment of miR-3085-5p inhibition promoted cell proliferation. In addition, the mRNA and protein levels of Col1A1 and α-SMA were reduced by miR-3085-5p overexpression, while the down-regulated miR-3085-5p showed opposite effects (Fig. S2b, c). Therefore, miR-3085-5p may down-regulate HSC activation. We further validated whether MIAT-mediated miR-3085-5p is involved in HSC activation. Interestingly, miR-3085-5p inhibitor blocked down the effects of MIAT inhibition on HSC activation, including HSC proliferation, α-SMA, and type I collagen (Fig. 5f, g). Conversely, upregulated α-SMA and type I collagen expression in HSC with MIAT upregulation were inhibited by miR-3085-5p mimics (Fig. 5h). Combined with these, our findings suggest that MIAT inhibition reduces HSC activation, at least partially, via sponging miR-3085-5p.

### MIAT regulates HSC activation through miR-3085-5p/YAP axis

**Fig. 6:**
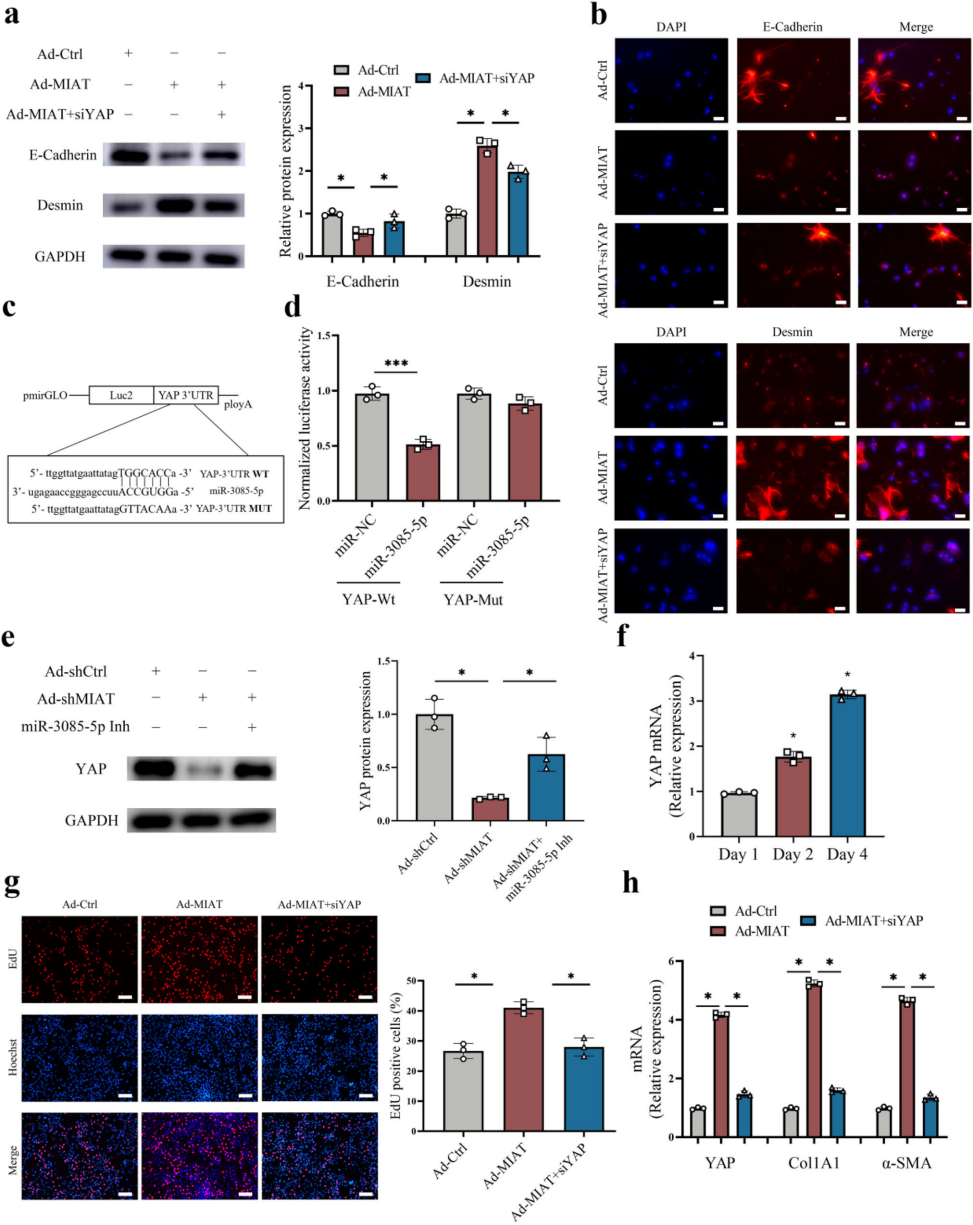
Loss of MIAT inhibits Hippo/EMT signaling pathway and HSC activation via YAP. **a** Protein expressions of E-cadherin and Desmin in HSCs transfected with Ad-Ctrl, Ad-MIAT or Ad-MIAT plus siYAP (*n* = 3 per group). **b** Immunofluorescence staining for E-cadherin and Desmin in HSCs transfected with Ad-Ctrl, Ad-MIAT or Ad-MIAT plus siYAP. Scale bar, 20 μm. **c** Binding sites of miR-3085-5p with respect to YAP. **d** Luciferase assays of pmirGLO-YAP-Wt or pmirGLO-YAP-Mut in HEK-293T cells with miR-NC or miR-3085-5p (*n* = 3 per group). **e** Protein expression of YAP in HSCs transfected with Ad-shCtrl, Ad-shMIAT or Ad-shMIAT plus miR-3085-5p inhibitor (*n* = 3 per group). **f** YAP expression in primary HSCs at day 1, 2 and 4 (*n* = 3 per group). **g** Cell proliferation in HSCs transfected with Ad-Ctrl, Ad-MIAT or Ad-MIAT plus siYAP (*n* = 3 per group). Scale bar, 50 μm. **h** YAP, Col1A1 and α-SMA expression in HSCs transfected with Ad-Ctrl, Ad-MIAT or Ad-MIAT plus siYAP (*n* = 3 per group). Each value is the mean ± SD of three independent experiments. *p < 0.05.

**Fig. 7:**
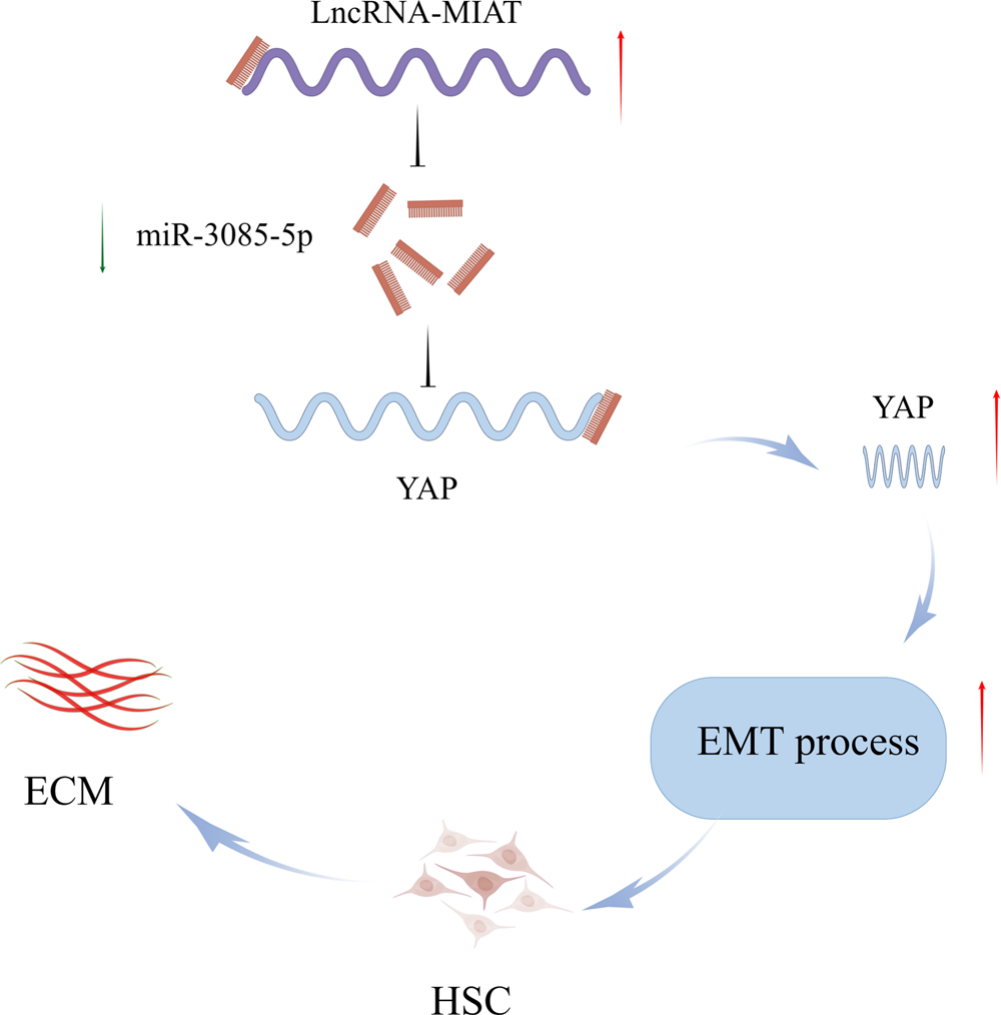
Schematic diagram of the biology function of MIAT in liver fibrosis. The figure was created by Figdraw.

It has been demonstrated that epithelial-to-mesenchymal transition (EMT) process is involved in HSC activation^13^. Immunoblot analysis showed that over-expression of MIAT accelerated EMT process, with reduced E-cadherin and increased Desmin (Fig. 6a). Immunofluorescence analysis further confirmed that E-cadherin protein was inhibited by MIAT while Desmin was enhanced by MIAT (Fig. 6b). Consistent with it, the expression of other EMT index (Vimentin and Snail) was also enhanced in cells with overexpressing MIAT (Fig. S3). Considering that miR-3085-5p was involved in the effects of loss of MIAT on HSC inactivation, we next explored the potential targets of miR-3085-5p. Increasing evidence demonstrated the crucial role of Hippo pathway-mediated EMT process in cancers and fibrotic diseases^14^. Bioinformatic analysis (miRDB) predicted that YAP, a member of Hippo pathway, may be a putative target of miR-3085-5p (Fig. 6c). As expected, luciferase reporter assays demonstrated that miR-3085-5p targeted YAP (Fig. 6d). The results of western blot showed that reduced YAP caused by MIAT knockdown in HSCs was attenuated by miR-3085-5p inhibitor (Fig. 6e). These data suggest YAP is a target of miR-3085-5p. In primary HSCs, YAP was gradually up-regulated during HSC activation (Fig. 6f), indicating that YAP expression is negatively correlated with miR-3085-5p level. In addition, overexpression of YAP promoted proliferation and collagen deposition potential, whereas YAP knockdown showed the opposite trends (Fig. S4). Therefore, YAP may be involved in the development of liver fibrosis. Then, the roles of YAP in MIAT-mediated HSC activation and EMT process were examined. Notably, loss of YAP contributed to inhibiting the effects of MIAT on HSC activation, including HSC proliferation, α-SMA, and Col1A1 (Fig. 6g, h). Furthermore, enhanced EMT process caused by MIAT was blocked down by loss of YAP (Figs. 6a, b and S3). Conversely, reduced EMT process and collagen deposition caused by MIAT knockdown were enhanced by over-expression of YAP (Fig. S5). Our data demonstrate that MIAT enhances HSC activation through miR-3085-5p/YAP/EMT axis (Fig. 7).

## Discussion

MIAT is considered as a risk factor in the progression of myocardial infarction. High expression of MIAT has been found in the blood samples from patients with atrial fibrillation^15^. It has been reported that MIAT could promote proliferation and invasion of HCC cells through sponging miR-214^16^. In this study, MIAT was confirmed as a pro-fibrotic factor in liver fibrosis. The expression of MIAT was increased in the liver tissues from both cirrhotic patients and CCl_4_ mice. HSC proliferation and collagen deposition were inhibited by Ad-shMIAT. Owing to the up-regulation of MIAT in liver fibrosis, the level of miR-3085-5p was reduced and its target YAP was increased, resulting in the enhancement of Hippo pathway-mediated EMT process in HSCs.

LncRNAs have been proven to be involved in many key biological functions, including cell proliferation, DNA damage, gene transcription, and epigenetic modifications. LncRNAs could play a regulatory role in human diseases via interacting with nuclear factors in the nucleus and/or acting as a competitive endogenous RNA (ceRNA)^17^. For example, MIAT promotes the proliferation and metastasis of colorectal cancer cells via miR-132/Derlin-1 pathway^18^. Liu et al. reported that MIAT knockdown potentiates the therapeutic effect of transcatheter arterial embolization in HCC by regulating the miR-203a/HIF-1α axis^19^. In this study, higher expression of MIAT was shown in the cytoplasm of HSCs. Results of RIP experiment showed that MIAT could act as a miRNA-binding platform. Further studies showed that MIAT was predicted to interact with miR-3085-5p, which was supported by luciferase reporter assays and pull-down assay. FISH experiment revealed the co-localization between MIAT and miR-3085-5p. miR-3085-5p, gradually decreased in the progression of liver fibrosis in vivo and in vitro, was negatively correlated with MIAT level. MIAT inhibition-reduced HSC proliferation and activation was attenuated by miR-3085-5p inhibitor. These data suggest that loss of MIAT may suppress liver fibrosis, at least in part, via sponging miR-3085-5p.

Hippo signaling pathway, a growth inhibition-related pathway, participates in carcinogenesis, tissue regeneration and functional regulation of stem cells^20^. When Hippo signaling pathway is inactivated, dephosphorylated YAP/TAZ interacts with transcription factors to promote growth-related gene expression, which finally mediates proliferation and migration^21^. In this study, YAP, a negative regulator in Hippo signaling pathway, was considered as a target of miR-3085-5p. MIAT knockdown-suppressed YAP was restored by miR-3085-5p inhibitor. EMT is a biological process that epithelial cells gradually lose epithelial features and acquire mesenchymal features^22^. EMT process plays a crucial role during organismal development, wound healing and tissue fibrosis. EMT process takes a part in many cancer cells for the processes of invasion and migration during cancer development^23^. Increasing evidence has shown the relation between MIAT and EMT. For example, MIAT has been shown to promote EMT-related biomarkers in renal fibrosis via targeting miR-145-3p/ EIF5A2 axis^10^. Zhong et al. found that MIAT induces the process of EMT via Wnt/β-catenin signaling pathway in tongue squamous cell carcinoma^24^. It is known that Hippo pathway-mediated EMT process is involved in cancers^25^. Huang et al. found that PDLIM1 knockdown-mediated EMT process contributes to YAP up-regulation in HCC^26^. In the present study, increased MIAT promoted EMT process, cell proliferation and collagen deposition in HSCs, which was inhibited by loss of YAP. Conversely, reduced EMT process and collagen deposition caused by MIAT knockdown was enhanced by over-expression of YAP. Therefore, MIAT promotes liver fibrosis progression via miR-3085-5p/YAP/EMT pathway.

Recently, lncRNAs have been found to act as promising biomarkers in the diagnosis of various human diseases. Previously, we found that lncRNA SNHG7 is a promising biomarker for chronic hepatitis B patients with liver fibrosis^27^. LncRNA-PDPK2P, an oncogene in HCC, could serve as a biomarker for the diagnosis, prognosis and therapy of HCC^28^. In this study, MIAT was highly expressed in the cirrhotic patients and positively related with fibrosis markers. ROC curve disclosed that MIAT exhibited a good performance in the diagnosis of liver fibrosis. Taken together, MIAT may be a potential biomarker for patients with liver fibrosis. Further studies with larger samples should be performed to verify the accuracy of MIAT in diagnosing liver fibrosis.

In conclusion, we demonstrate that MIAT-mediated HSC activation is, at least in part, via miR-3085-5p/YAP/EMT signaling pathway.

## Methods

### Ethnical statement

This study was approved by the research ethics committee of the First Affiliated Hospital of Wenzhou Medical University. All patients gave their informed consent. All methods of this study were carried out in accordance with the Declaration of Helsinki. The animal study was reviewed and approved by the Experimental Animal Center of Wenzhou Medical University. Wenzhou Medical University’s Animal Care and Use Committee approved all mice experiments in the Experimental Animal Center.

### Clinical samples collection

The First Affiliated Hospital of Wenzhou Medical University provided the liver tissues of 40 cirrhotic patients and 40 healthy volunteers who had partial liver resection or liver biopsy. The studies involving human participants were reviewed and approved by the Human Research Ethics Committee in the First Affiliated Hospital of Wenzhou Medical University. Informed consents were obtained from all participants before obtaining the liver tissues. Moreover, all liver samples were verified by the histopathological examination.

### Construction of CCl_4_ mice model

Twenty-four male C57BL/6J mice (6–8 weeks of age) were provided by the Experimental Animal Center of Wenzhou Medical University and randomly divided into the control (*n* = 6), CCl_4_ (*n* = 6), CCl_4_ combined with adenoviral vectors expressing scrambled shRNA (Ad-shCtrl) (*n* = 6) and CCl_4_ combined with Ad-shMIAT groups (*n* = 6). Mice in the CCl_4_ group were injected with 7 μl/g of 10% CCl_4_ in olive oil twice a week for 6 weeks. Equivalent volume of olive oil was treated in the control group. Besides, another two groups were treated with the 10% CCl_4_ with Ad-shCtrl for Ad-shCtrl group and 10% CCl_4_ with Ad-shMIAT for Ad-shMIAT group. Ad-shMIAT (1 × 10^9^ PFU/100 μl) was injected in a way of tail vein injection every 2 weeks for 6 weeks. Wenzhou Medical University’s Animal Care and Use Committee approved all experiments conducted on animals in the Experimental Animal Center. After the above procedure, mice were anesthetized, and their livers were used for further investigation.

### Measurement of hydroxyproline

After being homogenized by HCl, liver tissues were hydrolyzed at 120 °C. Then, sediments were removed after centrifugation with 12,000 × *g* at 4 °C for 10 min. The hydroxyproline colorimetric test kit (BioVision, San Francisco, CA, USA) was used to detect the level of hydroxyproline after the HCl were evaporated.

### Isolation and extraction of primary HSCs

Isolation of primary HSCs was performed as previously described^29^. Liver tissues were perfused and digested by multiple solution to prepare cell suspension. Primary HSCs were carried out via the density gradient centrifugation of the dispersed cell suspension. The quality of cultures reached >98%, which was verified by α-SMA immunocytochemical staining.

### Quantitative real-time PCR (qRT-PCR)

Total RNA was extracted from cells and liver tissues using Trizol Reagent. Then, RevertAid First Strand cDNA Synthesis Kit (Thermo Fisher Scientific) was used to reverse RNA into cDNA. The nuclear and cytoplasmic MIAT from HSCs was extracted by a PARIS Kit (Thermo Fisher Scientific, Waltham, MA, USA). The expressive levels of MIAT, Col1A1, α-SMA, miR-3085-5p, YAP, E-cadherin, Desmin, Vimentin and Snail were measured in the 7500 rapid quantitative PCR system with SYBR Green master mix (Applied Biosystems, USA). U6 served as an internal reference for miR-3085-5p. Glyceraldehyde-3-phosphate dehydrogenase (GAPDH) was used to normalize the relative levels of MIAT and mRNAs. The primer sequences were shown in Table S1.

### Cell transfection

GenePharma biotechnology (Shanghai, China) provided miR-3085-5p mimics, miR-3085-5p inhibitor and miR-negative control (miR-NC). In total, 100 nM miRNA was used for cell transfection. Ad-shMIAT, Ad-shCtrl, adenoviral vectors expressing MIAT (Ad-MIAT) and adenoviral vectors expressing a control scrambled sequence (Ad-Ctrl) were collected from Genomeditech.

### Cell proliferation assay

Cell Counting Kit-8 (CCK8) and EdU cell proliferation assay kit were used to analyze cell proliferation. HSCs (3000 cells/well) were seeded into 96-well plates in a volume of 100 μl of complete media. Each well received 10 μl of the CCK8 solution (Dojindo, Japan) at 0, 24, 48 and 72 h after seeding. Each well was measured at 450 nm in accordance with the manufacturer’s recommendation following 3 h incubation. BeyoClick™ EdU Cell Proliferation Kit with Alexa Fluor 488 was used for cell proliferation analysis. After marking with EdU for 2 h, primary HSCs were fixed with formalin and permeated with 0.3% Triton X-100. Cells were then incubated in the dark for 30 min with Click Reaction Mixture, and stained with Hoechst. The incorporation cells quantity of EdU was calculated after visualization processing.

### Immunofluorescence

After cells were growth in cover glasses, 4% paraformaldehyde was then used to fix cells for 15 min and 0.3% Triton X-100 was performed to permeabilize cells. Non-specific binding sites of cells were blocked by 5% goat serum for 1 h at 37 °C. Then, cells were incubated with the following primary antibodies overnight at 4 °C: anti-E-cadherin antibody, anti-Desmin antibody, anti-α-SMA antibody, and anti-type I collagen antibody. On the next day, fluorescein-labeled secondary antibody was incubated at 37 °C for 1 h (1:50 dilution). Besides, nucleus was stained by 4,6-diamidino-2-phenylindole (DAPI) in the dark and photographed by fluorescence microscopy.

### RIP assay

According to the manufacturer’s instructions, the EZ-Magna RIP kit (Millipore) was used for the RIP assay. Briefly, lysed primary HSCs were immunoprecipitated with anti-Ago2 antibody (Abcam) and the corresponding anti-IgG at 4 °C overnight. Finally, MIAT level was analyzed by qRT-PCR.

### Luciferase reporter assay

Oligonucleotide containing MIAT sequence was constructed and cloned into pmirGLO-MIAT dual-luciferase reporter vectors. The pmirGLO-MIAT dual-luciferase reporter vector was co-transfected with miR-3085-5p or miR-NC into HEK293T cells via lipofectamine technology. The Dual-Luciferase Assay Kit (Promega, Madison, WI, USA) was used to examine relative luciferase activity after 48 h. Renilla luciferase was performed as an internal reference and used for normalization.

### RNA pull-down assay

Bio-miR-3085-5p-Wt, bio-miR-3085-5p-Mut together with bio-miR-NC were produced from GenePharma (Shanghai, China). Biotin pull-down was performed according to the manufacturer’s instructions. In brief, miR-3085-5p-Mut, miR-3085-5p-Wt and miR-NC were labeled with biotin and transfected into cells for 48 h. After cells were lysed by lysis buffer for 10 min, streptavidin beads were incubated with the lysates for 4 h at 4 °C. Then, the co-immunoprecipitated RNAs were washed for 5 times with binding and wash buffer. TRIzol reagent was used to extract bound RNAs for qRT-PCR analysis.

### FISH

Dig-labeled miR-3085-5p probes and biotin-labeled specific to MIAT probes were used for the double FISH assay. Besides, the nuclei were counterstained by DAPI. The observation of fluorescence signals was detected by the confocal laser microscope system (Leica, Wetzlar, Germany).

### Western blot

Total protein of cells was extracted by RIPA buffer (Thermo Fisher Scientific) with protease and phosphatase inhibitors (Thermo Fisher Scientific). We measured total protein concentration using a BCA protein assay kit. In total, 10% sodium dodecyl sulfate-polyacrylamide gel electrophoresis was used to separate the proteins. Then the electrophoresed proteins were transferred to the polyvinylidene fluoride membrane. Primary antibodies were incubated with the membrane at 4 °C overnight after blocking the non-specific sites with 5% skim milk for 2 h at room temperature. Primary antibodies were included anti-YAP (cat no. 13584-1-AP; 1:2000; Proteintech), anti-E-cadherin (cat No. 20874-1-AP; 1:5000; Proteintech), anti-Desmin (cat no. ab32362; 1:2000; abcam), anti-Vimentin (cat no. ab92547; 1:1000; abcam), anti-Snail (cat no. 13099-1-AP; 1:1000; Proteintech), anti-Type I collagen (cat no. ab260043; 1:1000; abcam), anti-α-SMA (cat no. ab32575; 1:1000; abcam) and anti-GAPDH (cat no. ab8245; 1:1000; abcam). On the next day, the membrane was incubated with secondary antibody for 1 h at room temperature. GAPDH acted as an internal reference.

### Statistics and reproducibility

These experiments were independently repeated at least three times, and all results were shown as mean ± standard deviation (SD). Unpaired two-tailed Student’s *t* test was applied to compare the differences between two groups. One-way ANOVA was used to analyze the comparisons between multiple groups. The correlation between two groups was analyzed by Pearson correlation analysis. ROC curve was created for the evaluation of MIAT in cirrhotic patients. Sample size and replicates are stated in the corresponding figure legends. The statistical analyses were conducted using SPSS 21.0 software (SPSS Inc., Chicago, IL, USA) or GraphPad Prism 8 (Graph-Pad Software Inc., La Jolla, CA, USA). *p* < 0.05 was considered statistically significant.

### Reporting summary

Further information on research design is available in the Nature Portfolio Reporting Summary linked to this article.

### Supplementary information

Supplementary Material

Description of Additional Supplementary Files

Supplementary Data 1

Reporting Summary

## Data Availability

The source data for the graphs in the main figures are provided in Supplementary Data 1. The original uncropped blot/gel images of the main figures are provided in Fig. S6. Figure 7 was created by Figdraw (grant ID: IOWIPcb189). The other data supporting the findings of this study are available upon request from the corresponding author.
