# Author Correction: LncRNA-MIAT activates hepatic stellate cells via regulating Hippo pathway and epithelial-to-mesenchymal transition

**DOI:** 10.1038/s42003-023-05712-2

**Published:** 2024-01-02

**Authors:** Yating Zhan, Qiqi Tao, Qishan Meng, Rongrong Zhang, Lifan Lin, Xinmiao Li, Lei Zheng, Jianjian Zheng

**Affiliations:** 1 Key Laboratory of Diagnosis and Treatment of Severe Hepato-Pancreatic Diseases of Zhejiang Province, The First Affiliated Hospital of Wenzhou Medical University, Wenzhou, 325000 China; 2 Key Laboratory of Clinical Laboratory Diagnosis and Translational Research of Zhejiang Province, The First Affiliated Hospital of Wenzhou Medical University, Wenzhou, 325000 China; 3 Laboratory Medicine Center, Nanfang Hospital, Southern Medical University, Guangzhou, 510515 China

**Keywords:** Mechanisms of disease, Cell death

Correction to: *Communications Biology* 10.1038/s42003-023-04670-z, published online 18 March 2023.

The original version of the Article contained errors in Fig. 6g arising from the authors using the incorrect images for the EdU and Hoechst staining (including the merge) in the middle panel (“Ad-MIAT”) of the Figure. This has now been corrected in the PDF and HTML versions of the Article.

