# Supplementary material for: LncRNA-MIAT activates hepatic stellate cells via regulating Hippo pathway and epithelial-to-mesenchymal transition
Source: Commun Biol. 2023 Mar 18;6:285. doi: 10.1038/s42003-023-04670-z (PMC10024685; doi:10.1038/s42003-023-04670-z)
Supplement: Supplementary file 1 — Supplementary Material [file 42003_2023_4670_MOESM1_ESM.pdf]

Table S1

The gene primer sequence used for qRT-PCR.

Gene	Forward (5'-3')	Reverse (5'-3')
MIAT (human)	CACTGCTCCTGGATTCTGTTCTTGG	AACGCTGGGACTGTCTCCTCTG
MIAT (mouse)	TTTGCCTTTCTGGTCTGTTCCTTCC	CCGCCATCATCCAAGCCGTTAG
Col1A1 (human)	CCCCGAGGCTCTGAAGGT	GCAATACCAGGAGCACCATTG
Col1A1 (mouse)	CGATGGATTCCCGTTCGAGT	GAGGCCTCGGTGGACATTAG
α-SMA (human)	GTTCCGCTCCTCTCTCCAAC	GTGCGGACAGGAATTGAAGC
α-SMA (mouse)	TCTTCCAGCCATCTTTCATTGGGAT	CCTGTTTTGGCTCCCTATGTCT
miR-3085-5p (human)	AACCACTAGGTGCCATTCTGAGG	ATCCAGTGCAGGGTCCGAGG
miR-3085-5p (mouse)	AACCGGAGGTGCCATTCCGA	ATCCAGTGCAGGGTCCGAGG
YAP (mouse)	ATTTCGGCAGGCAATACGGA	TGCGCAGAGCTAATTCCTGA
E-cadherin (mouse)	ATGTCCTGGGCAGAGTGAGA	TGGAGCTTTAGATGCCGCTT
Desmin (mouse)	CAGAGGCTCAAGGCCAAACTA	AGGGATTCGATTCTGCGCTC
Vimentin (mouse)	AGACCAGAGATGGACAGGTGA	CTGGTACTGCACTGTTGCAC
Snail (mouse)	TAGGTCGCTCTGGCCAACAT	CTGGAAGGTGAACTCCACACA
GAPDH (human)	AAGCCTGCCGGTGACTAAC	CGCCCAATACGACCAAATCAGA
GAPDH (mouse)	AGGAGAGTGTTTCCTCGTCC	TGAGGTCAATGAAGGGGTCG
U6 (human)	AGAGAAGATTAGCATGGCCCCTG	CAGTGCAGGGTCCGAGGT
U6 (mouse)	GAAGATTTAGCATGGCCCCTGC	CAGTGCAGGGTCCGAGGT

Fig. S1 Effect of MIAT on cell migration of HSCs.

(a) Wound healing assay. Scale bar, 200 μm. (b) Statics analysis of wound healing assay (n=3 per group). Each value is the mean ± SD of three independent experiments. **P*<0.05 compared with the control.

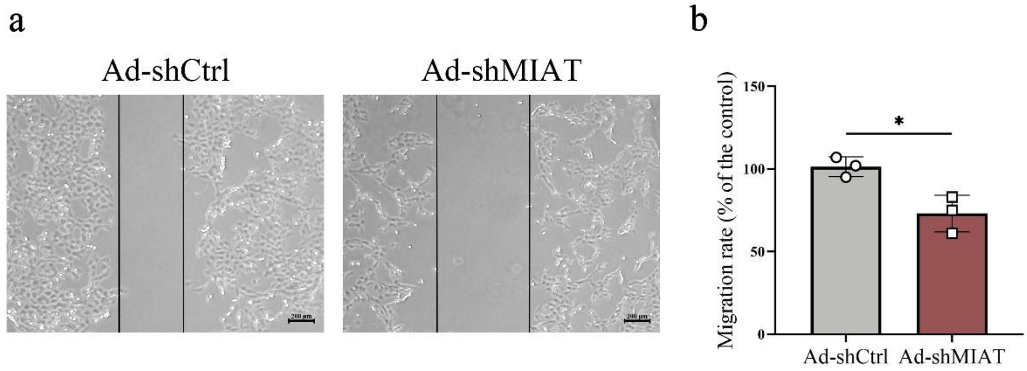

Fig. S2 The roles of miR-3085-5p in the activation of HSCs.

(a) CCK8 assay in HSCs with miR-3085-5p mimics or miR-3085-5p inhibitor treatment (n=3 per group). (b) miR-3085-5p, Col1A1 and α-SMA expression in HSCs with miR-3085-5p mimics or miR-3085-5p inhibitor treatment (n=3 per group). (c) Protein expressions of Type I collagen and α-SMA in HSCs with miR-3085-5p mimics or miR-3085-5p inhibitor treatment (n=3 per group). Each value is the mean ± SD of three independent experiments. *P<0.05, **P < 0.01 compared with the control.

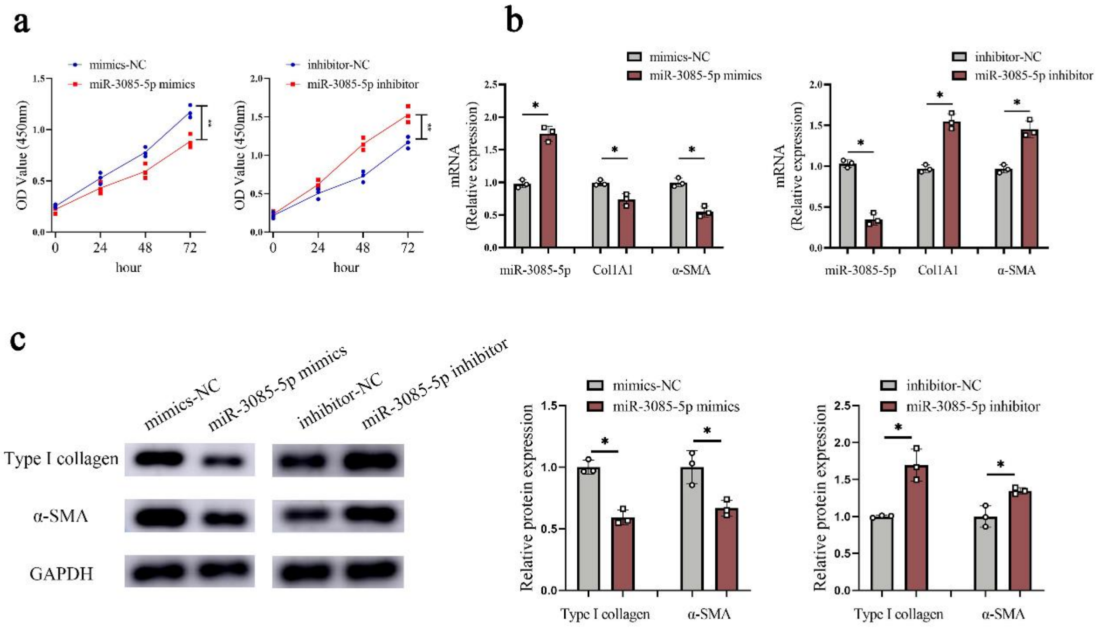

Fig. S3 MIAT enhances EMT process of HSCs via YAP.

(a) Vimentin and Snail expression in HSCs transfected with Ad-Ctrl, Ad-MIAT or Ad-MIAT plus siYAP (n=3 per group). (b) Protein expressions of Vimentin and Snail in HSCs transfected with Ad-Ctrl, Ad-MIAT or Ad-MIAT plus siYAP (n=3 per group). Each value is the mean ± SD of three independent experiments. **P*<0.05.

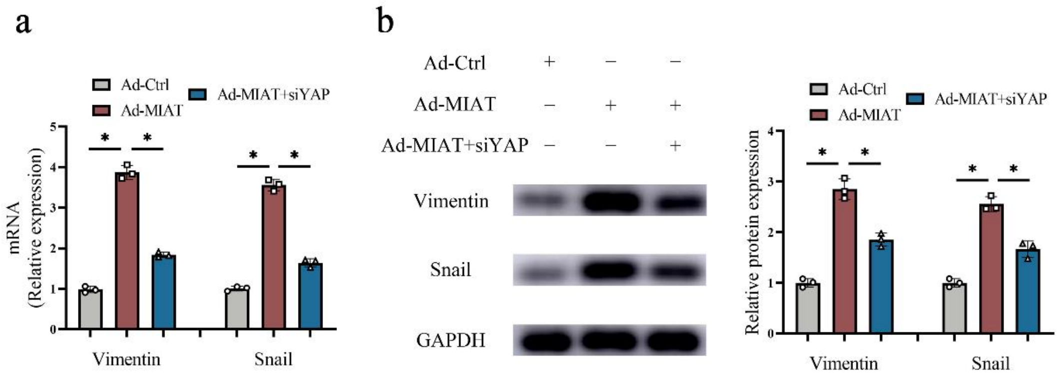

Fig. S4 The roles of YAP in the activation of HSCs.

(a) CCK8 assay in HSCs with siYAP or YAP treatment (n=3 per group). (b) YAP, Col1A1 and α-SMA expression in HSCs with siYAP or YAP treatment (n=3 per group). (c) Protein expressions of YAP, Type I collagen and α-SMA in HSCs with siYAP or YAP treatment (n=3 per group). Each value is the mean ± SD of three independent experiments. *P<0.05, **P < 0.01 compared with the control.

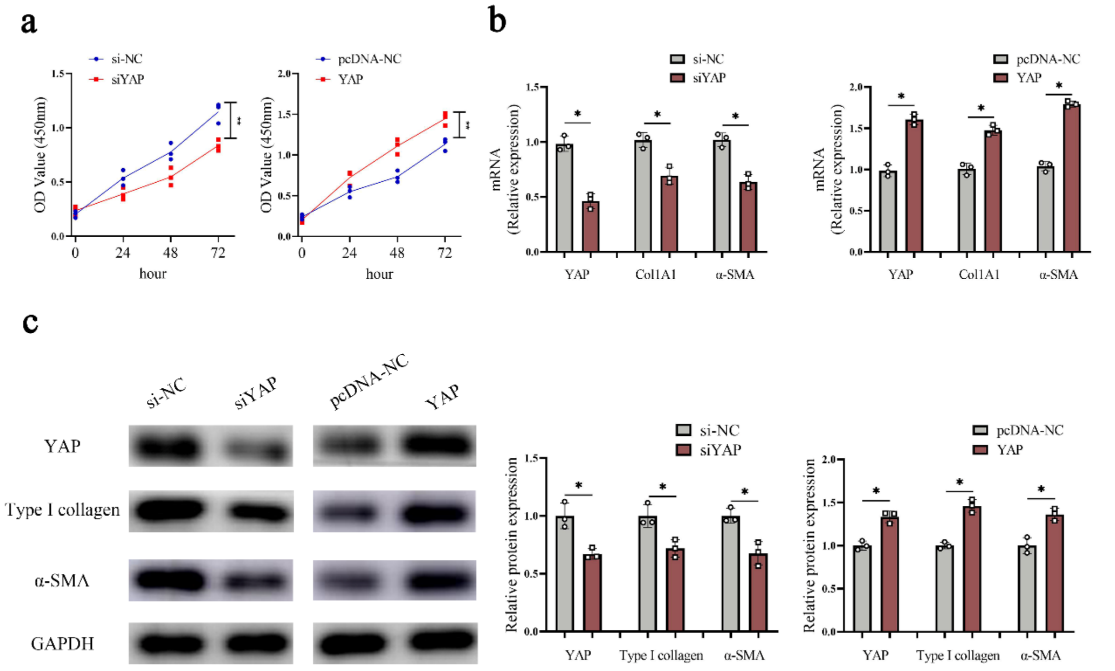

Fig. S5 Loss of MIAT inhibits Hippo/EMT signaling pathway and HSC activation via YAP.

(a) CCK8 assay in HSCs transfected with Ad-shCtrl, Ad-shMIAT or Ad-shMIAT plus YAP (n=3 per group). (b) E-Cadherin, Desmin, Col1A1 and α-SMA expression in HSCs transfected with Ad-shCtrl, Ad-shMIAT or Ad-shMIAT plus YAP (n=3 per group). (c) Protein expressions of E-Cadherin, Desmin, Type I collagen and α-SMA in HSCs transfected with Ad-shCtrl, Ad-shMIAT or Ad-shMIAT plus YAP (n=3 per group). Each value is the mean ± SD of three independent experiments. *P<0.05 and **P < 0.01.

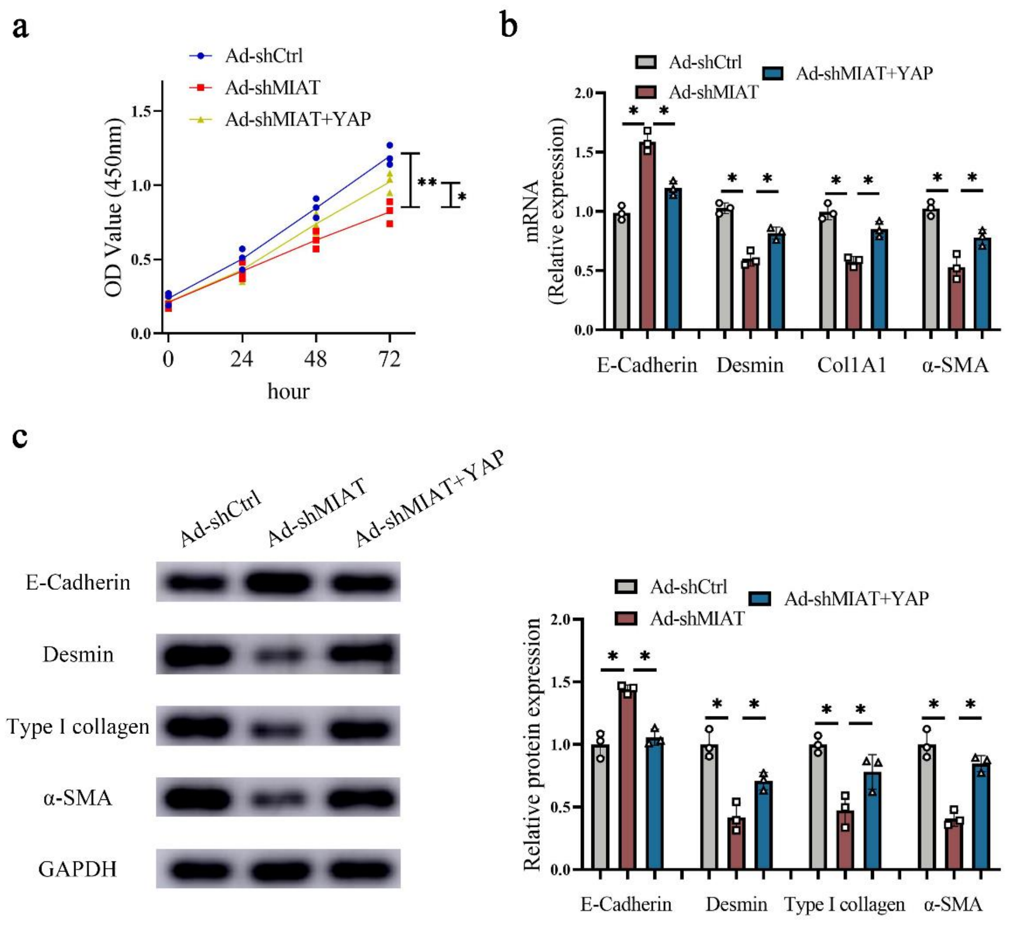

Fig. S6 Unedited blot/gel images

Fig. 5g

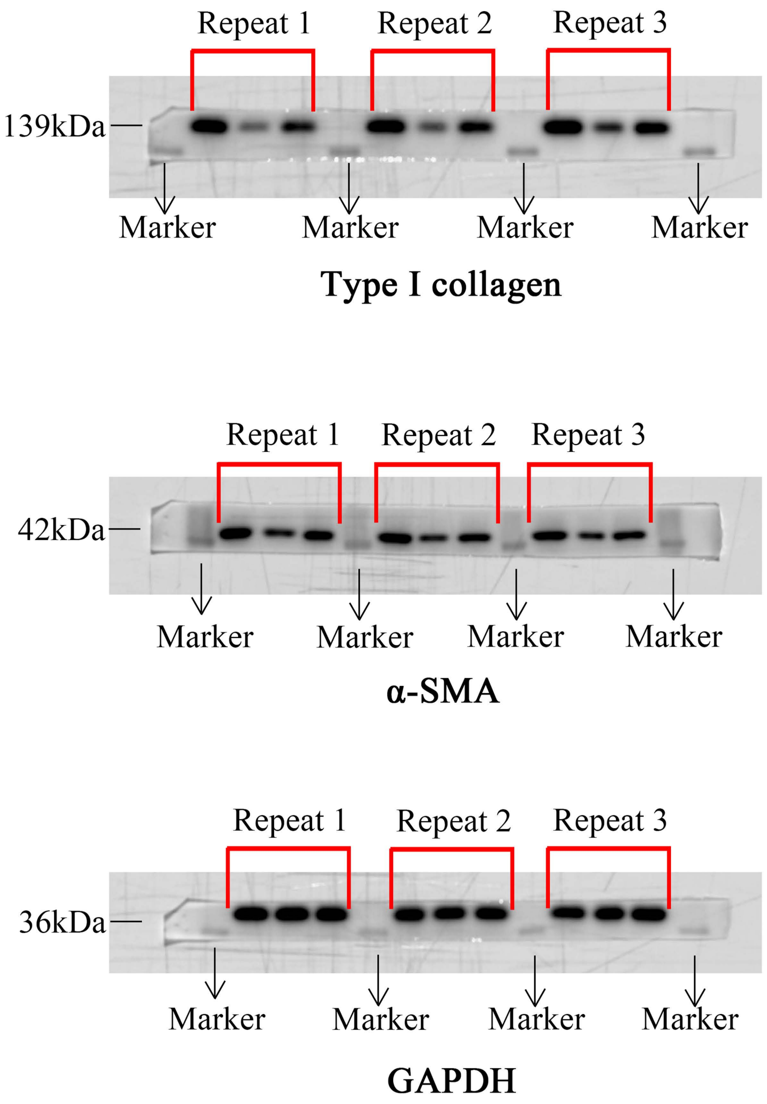

Fig. 5h

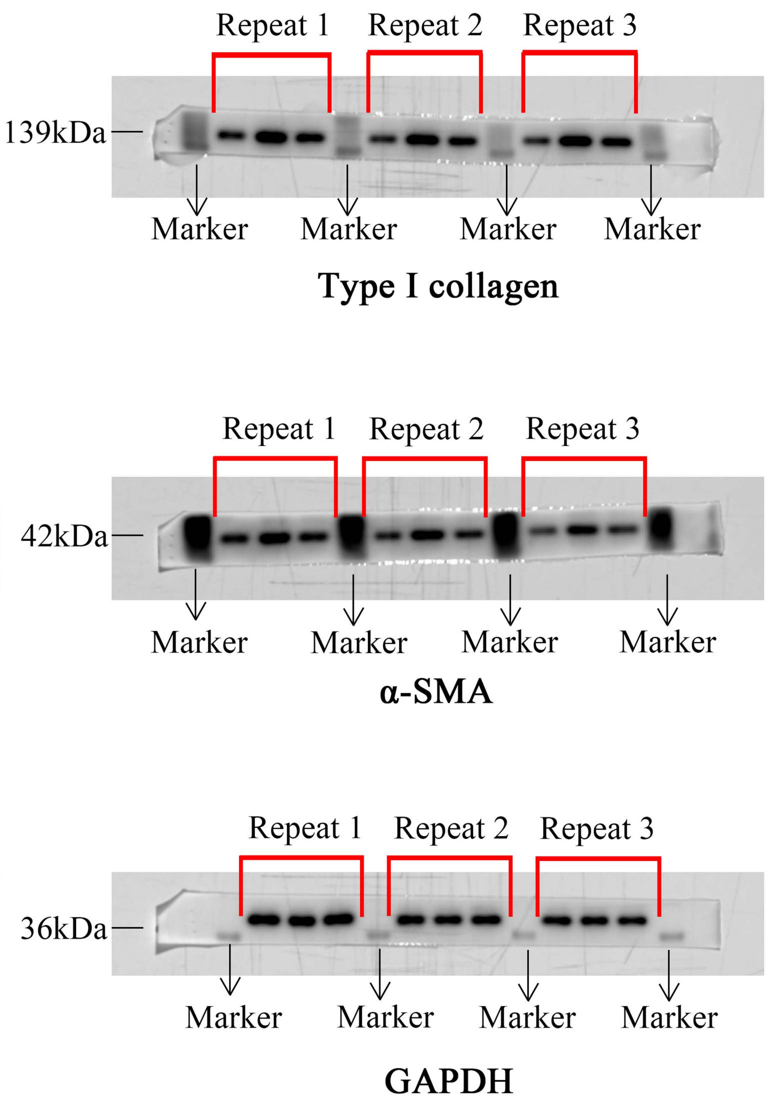

Fig. 6a

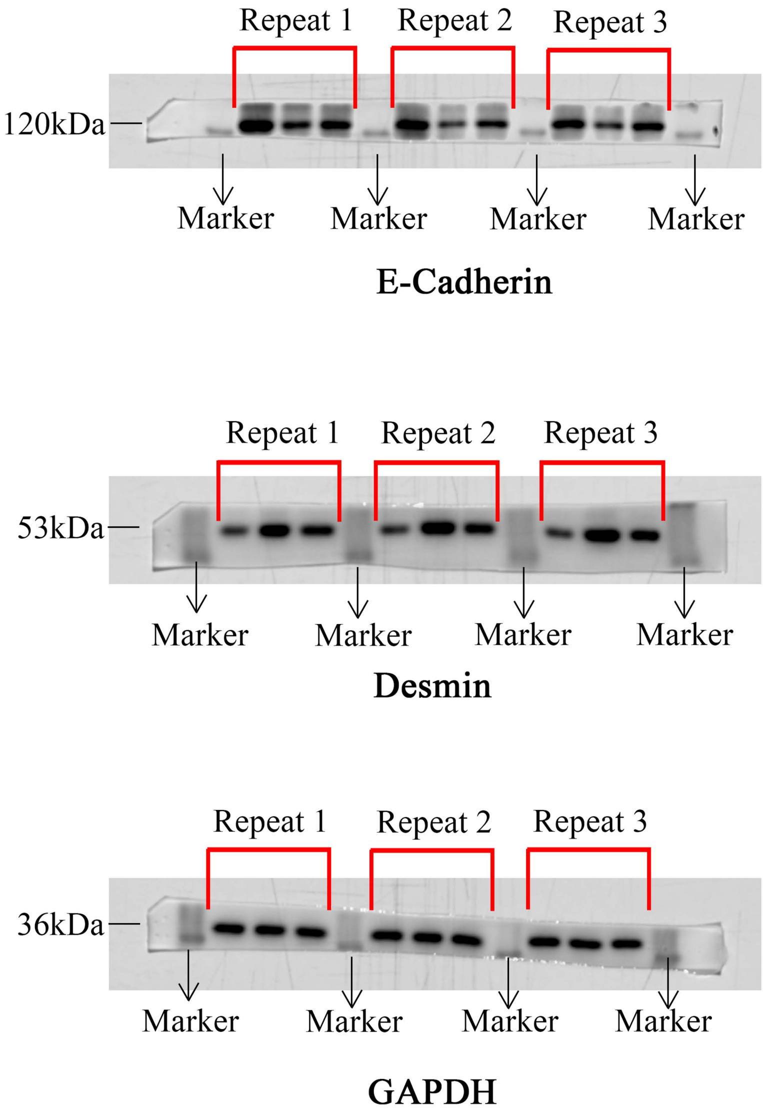
Repeat 1
Repeat 2
Repeat 3
120kDa
Marker
Marker
Marker
Marker
E-Cadherin
Repeat 1
Repeat 2
Repeat 3
53kDa
Marker
Marker
Marker
Marker
Desmin
Repeat 1
Repeat 2
Repeat 3
36kDa
Marker
Marker
Marker
Marker
GAPDH

Fig. 6e

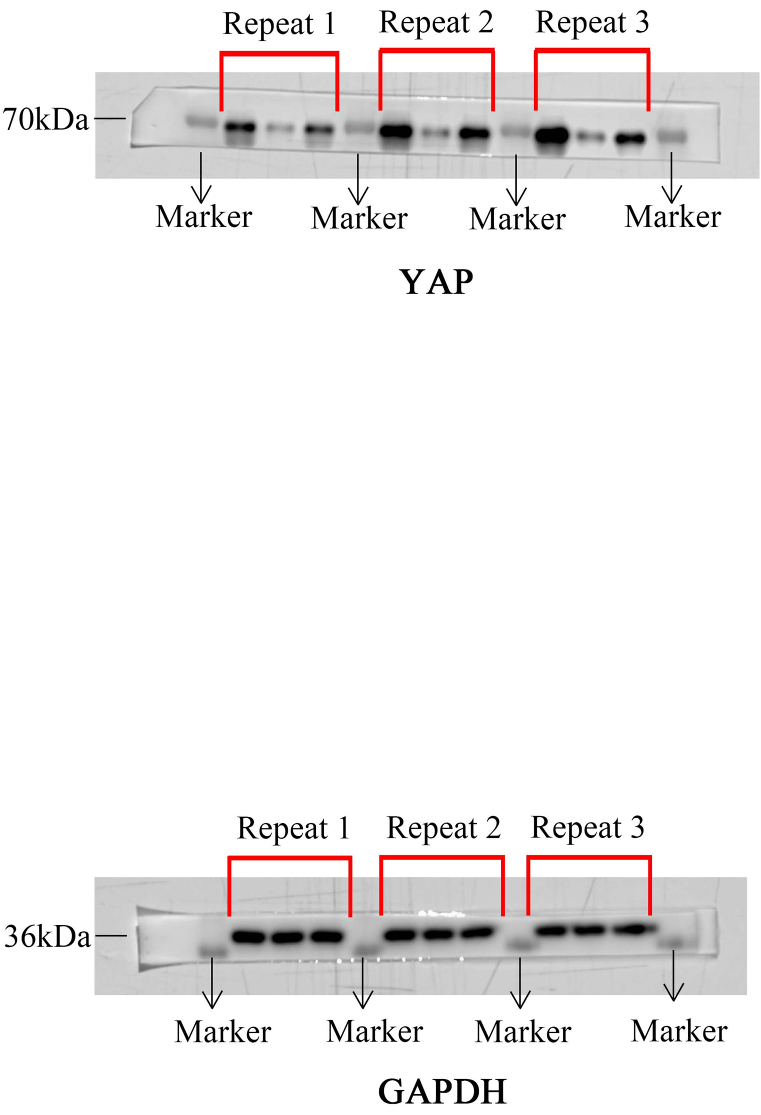
Repeat 1
Repeat 2
Repeat 3
70kDa
Marker
Marker
Marker
Marker
YAP
Repeat 1
Repeat 2
Repeat 3
36kDa
Marker
Marker
Marker
Marker
GAPDH

Fig. S2c

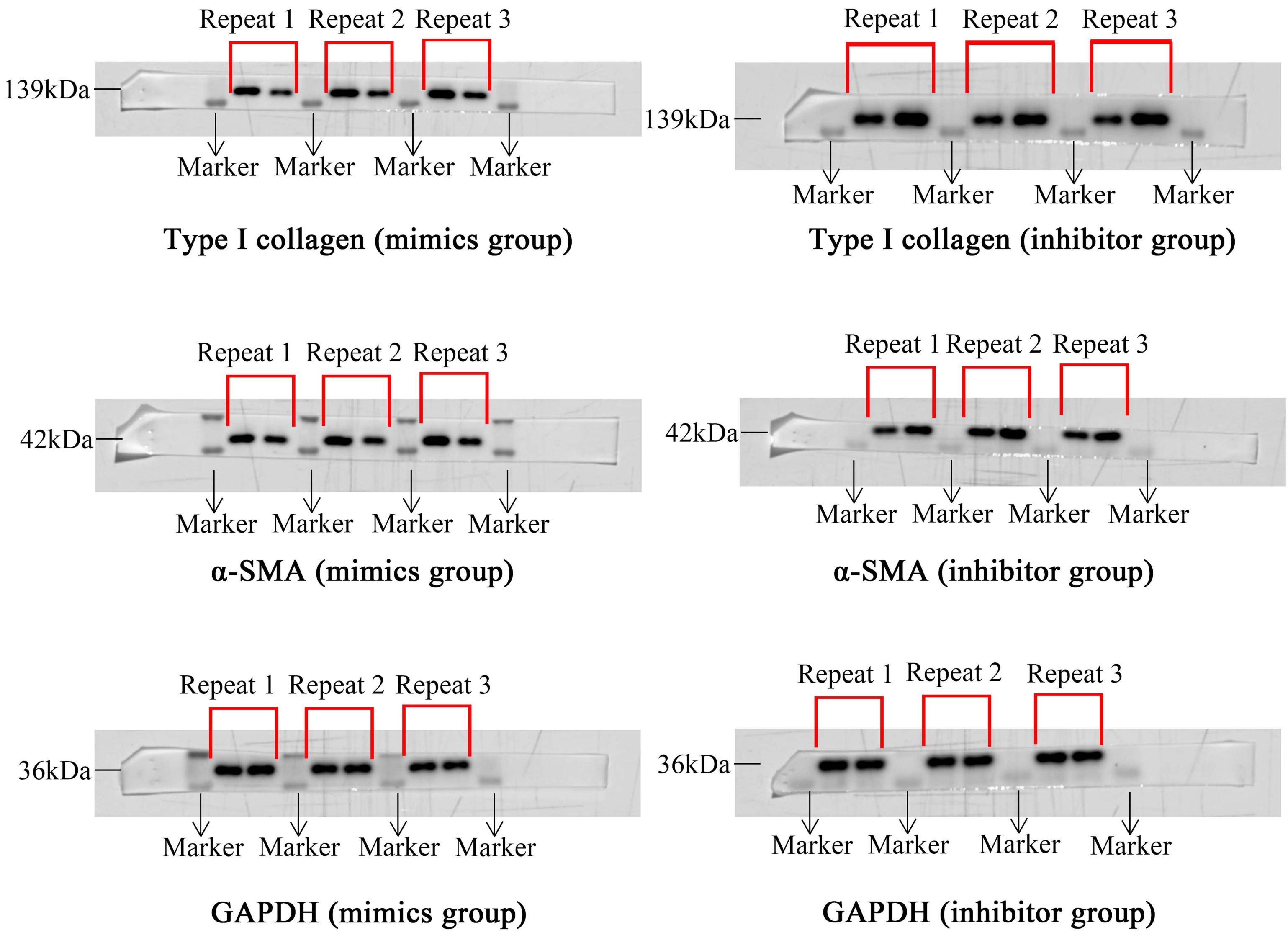

Repeat 1
Repeat 2
Repeat 3
139kDa
Marker
Marker
Marker
Marker
Type I collagen (mimics group)
Repeat 1
Repeat 2
Repeat 3
139kDa
Marker
Marker
Marker
Marker
Type I collagen (inhibitor group)
Repeat 1
Repeat 2
Repeat 3
42kDa
Marker
Marker
Marker
Marker
α-SMA (mimics group)
Repeat 1
Repeat 2
Repeat 3
42kDa
Marker
Marker
Marker
Marker
α-SMA (inhibitor group)
Repeat 1
Repeat 2
Repeat 3
36kDa
Marker
Marker
Marker
Marker
GAPDH (mimics group)
Repeat 1
Repeat 2
Repeat 3
36kDa
Marker
Marker
Marker
Marker
GAPDH (inhibitor group)

Fig. S3b

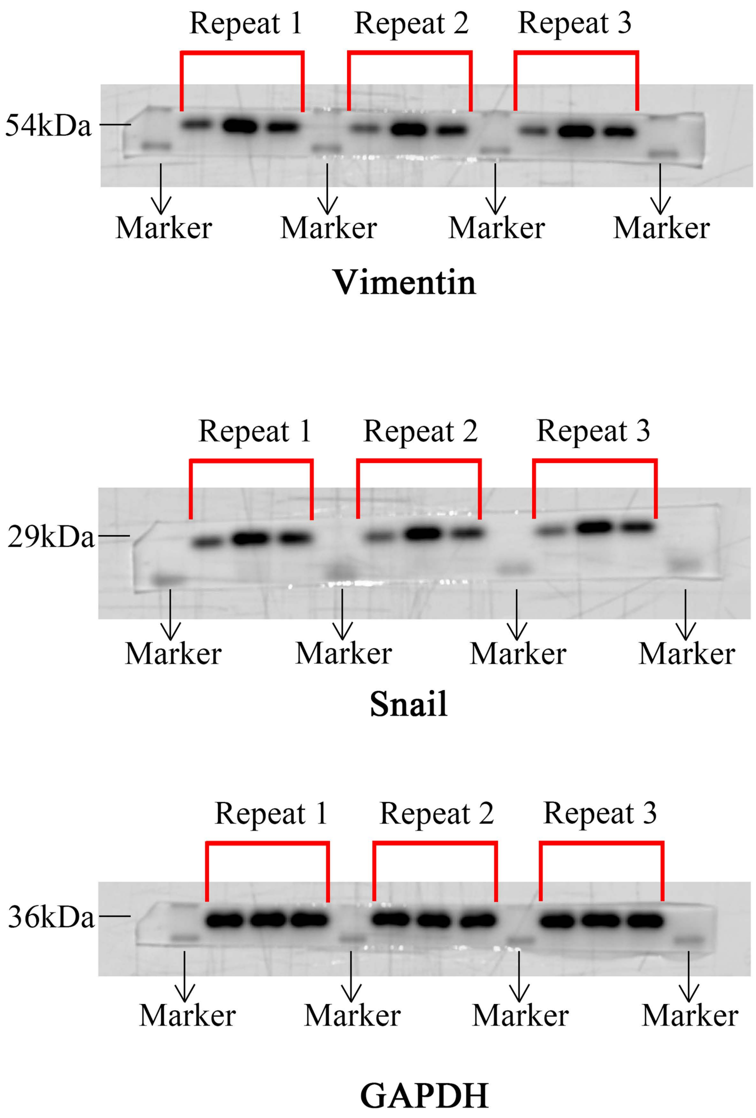
Repeat 1
Repeat 2
Repeat 3
54kDa
Marker
Marker
Marker
Marker
Vimentin
Repeat 1
Repeat 2
Repeat 3
29kDa
Marker
Marker
Marker
Marker
Snail
Repeat 1
Repeat 2
Repeat 3
36kDa
Marker
Marker
Marker
Marker
GAPDH

Fig. S4c

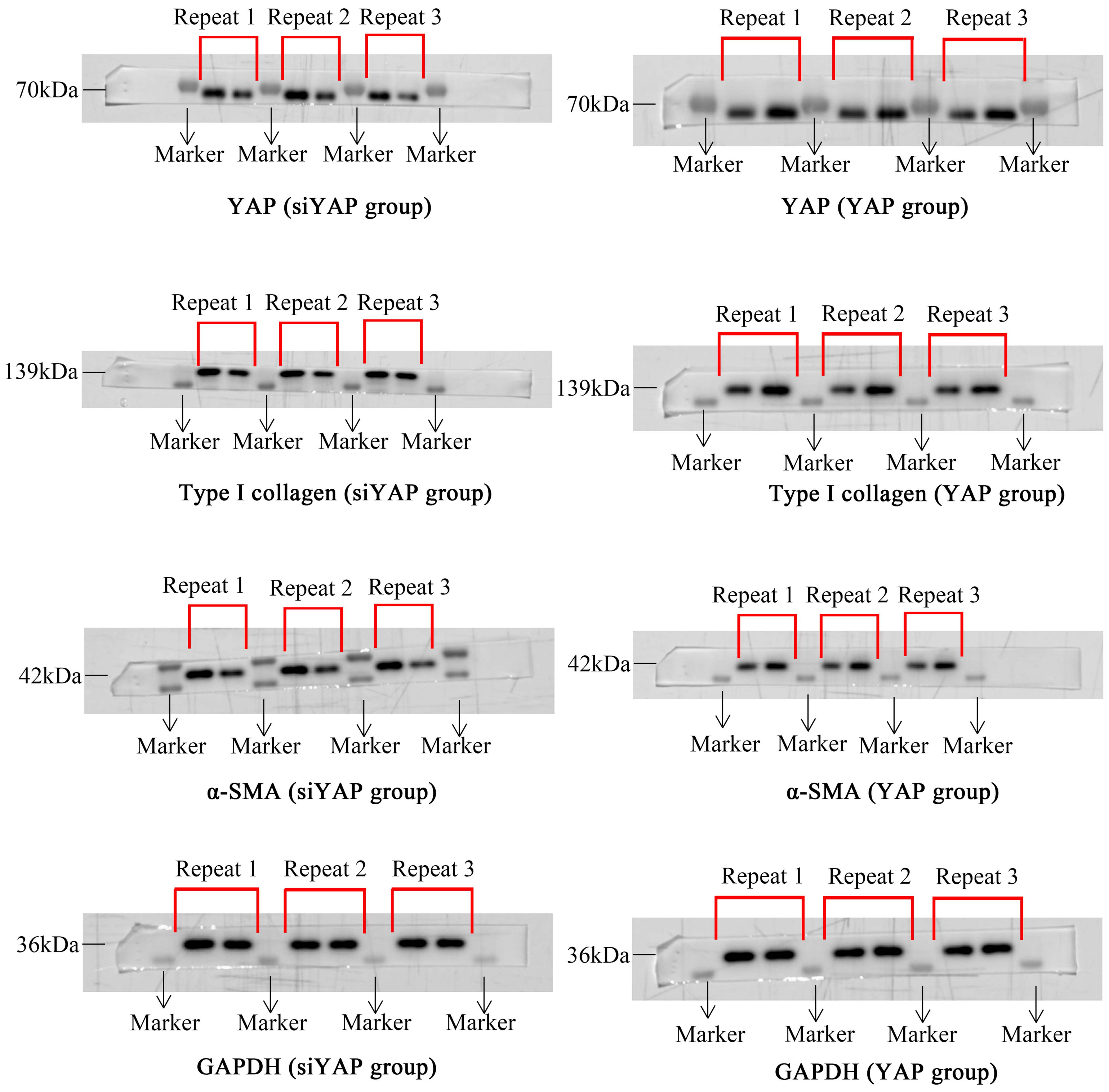

Repeat 1
Repeat 2
Repeat 3
70kDa
Marker
Marker
Marker
Marker
YAP (siYAP group)
Repeat 1
Repeat 2
Repeat 3
70kDa
Marker
Marker
Marker
Marker
YAP (YAP group)
Repeat 1
Repeat 2
Repeat 3
139kDa
Marker
Marker
Marker
Marker
Type I collagen (siYAP group)
Repeat 1
Repeat 2
Repeat 3
139kDa
Marker
Marker
Marker
Marker
Type I collagen (YAP group)
Repeat 1
Repeat 2
Repeat 3
42kDa
Marker
Marker
Marker
Marker
α-SMA (siYAP group)
Repeat 1
Repeat 2
Repeat 3
42kDa
Marker
Marker
Marker
Marker
α-SMA (YAP group)
Repeat 1
Repeat 2
Repeat 3
36kDa
Marker
Marker
Marker
Marker
GAPDH (siYAP group)
Repeat 1
Repeat 2
Repeat 3
36kDa
Marker
Marker
Marker
Marker
GAPDH (YAP group)

Fig. S5c

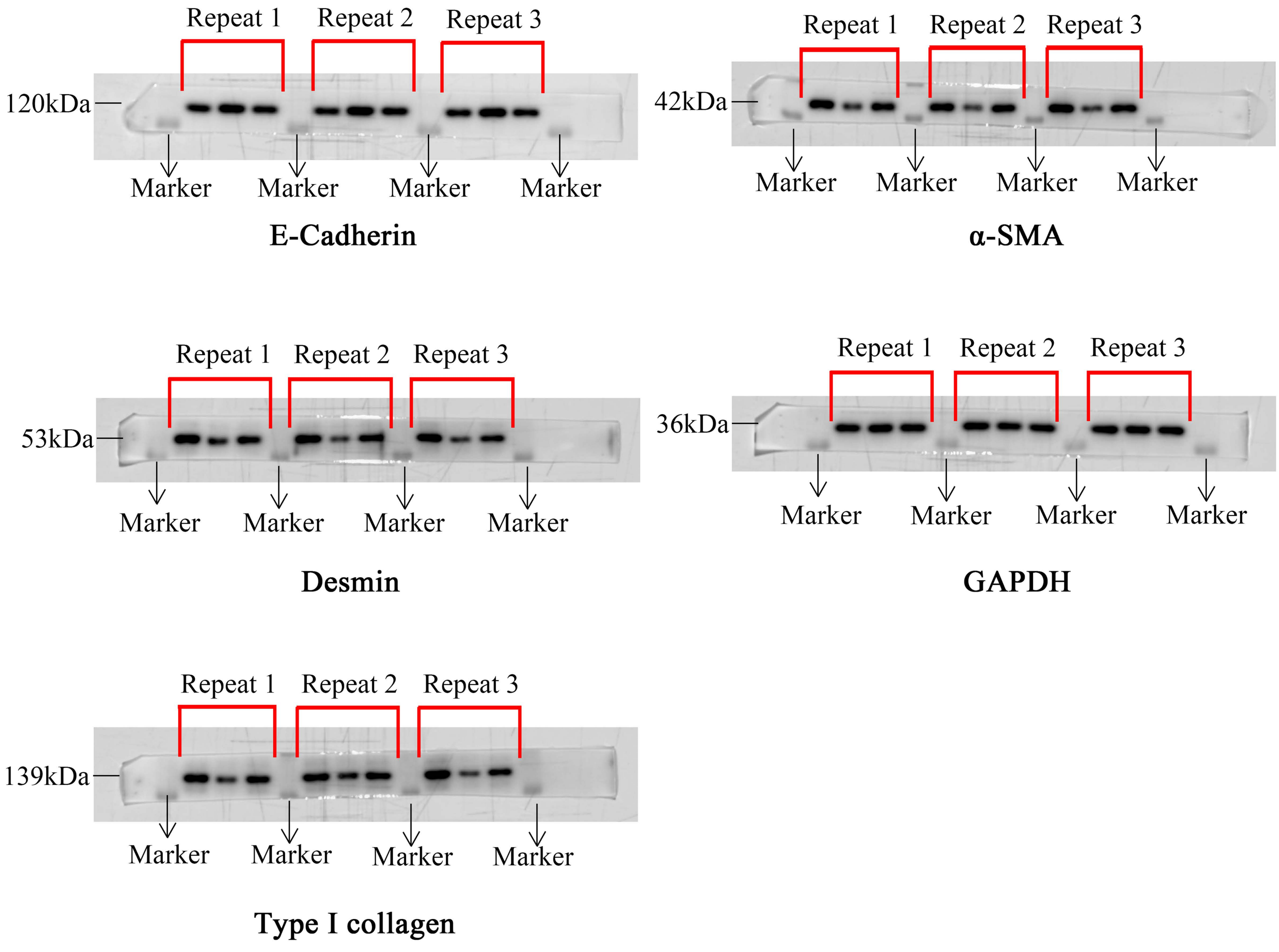
Repeat 1
Repeat 2
Repeat 3
120kDa
Marker
Marker
Marker
Marker
E-Cadherin
Repeat 1
Repeat 2
Repeat 3
42kDa
Marker
Marker
Marker
Marker
α-SMA
Repeat 1
Repeat 2
Repeat 3
53kDa
Marker
Marker
Marker
Marker
Desmin
Repeat 1
Repeat 2
Repeat 3
36kDa
Marker
Marker
Marker
Marker
GAPDH
Repeat 1
Repeat 2
Repeat 3
139kDa
Marker
Marker
Marker
Marker
Type I collagen
