# Supplementary material for: LncRNA-MIAT activates hepatic stellate cells via regulating Hippo pathway and epithelial-to-mesenchymal transition
Source: Commun Biol. 2023 Mar 18;6:285. doi: 10.1038/s42003-023-04670-z (PMC10024685; doi:10.1038/s42003-023-04670-z)
Supplement: Supplementary file 2 — Description of Additional Supplementary Files [file 42003_2023_4670_MOESM2_ESM.docx]

**Description of Additional Supplementary Files**

**File name:** Supplementary Data 1

**Description:** The source data for the graphs in the main figures.
